# Patient-derived lymphoblastoid cell lines harboring mitochondrial DNA mutations as tool for small molecule drug discovery

**DOI:** 10.1186/s13104-018-3297-6

**Published:** 2018-03-27

**Authors:** Randall Marcelo Chin, Tadas Panavas, Jeffrey M. Brown, Krista K. Johnson

**Affiliations:** 1 Alexion Pharmaceuticals, Inc., 100 College Street, New Haven, CT 06510 USA; 2 Biotherapeutic Molecule Discovery, Boehringer Ingelheim Pharmaceuticals, 900 Ridgebury Road, Ridgefield, CT 06877 USA; 3 Wave Life Sciences, 733 Concord Ave., Cambridge, MA 02138 USA

**Keywords:** Mitochondria, Idebenone, Respiration, Mitochondrial disease, ND1, ND4, ATP6, Lymphoblastoid cell lines, High-throughput screening

## Abstract

**Objective:**

Mitochondrial diseases are a group of devastating disorders for which there is no transformative cure. The majority of therapies for mitochondrial disease—approved, previously tested, or currently in development—are small molecules. The implementation of better cell-based models of mitochondrial disease can accelerate and improve the accuracy of small molecule drug discovery. The objective of this study is to evaluate the use of patient-derived lymphoblastoid cell lines for small molecule research in mitochondrial disease.

**Results:**

Five lymphoblastoid cell lines derived from mitochondrial disease patients harboring point mutations in mtND1, mtND4, or mtATP6 were characterized in two high throughput assays assessing mitochondrial function. In a pilot “clinical trial in a dish” experiment, the efficacy of idebenone—an approved therapy for mitochondrial disease—on the lymphoblastoid cell lines was tested. Idebenone increased the basal respiration of all lymphoblastoid cell lines except those harboring the 8993T>G point mutation in mtATP6. Our results posit lymphoblastoid cell lines as a strong model for mitochondrial disease research with small molecules and have implications for the clinical efficacy of idebenone.

## Introduction

Mitochondrial diseases are a group of clinically heterogeneous disorders that affect about one in 5000 people [1], and can be caused by mutations in the mitochondrial DNA (mtDNA). There is currently no cure for mitochondrial disease.

Lymphoblastoid cell lines (LCLs) are patient-derived cells resulting from the transformation of peripheral B lymphocytes by Epstein–Barr virus. There are many advantages to working with this cell model of mitochondrial disease. First, LCLs are relatively abundant and publically available, as many biorepositories collect, store, and distribute LCLs. Second, LCLs are effectively immortalized, and contain and replicate the mtDNA mutations found in mitochondrial disease patients. Third, the mitochondria of LCLs are robust, generally have a higher respiratory rate than that of cybrids [2], making them more sensitive to mitochondrial assays. Fourth, LCLs respond to mitochondrial inhibitors and other small molecules [3]. Lastly, the wide availability of LCLs derived from apparently healthy individuals can be used to find an average readout for “normal” control cell lines.

Because of their availability, immortality, and responsiveness, LCLs are a good screening tool for small molecule research. For example, once a library of LCLs from hundreds of patients is obtained, “clinical trial in a dish” studies can be done to test for drug efficacy in cells with a variety of nuclear and mitochondrial DNA. This could lead to the development of more broadly applicable therapies that are not biased towards any particular genotype. However, there are little to no studies on this topic. In this paper, we present results from five LCLs obtained from mitochondrial disease patients harboring disease associated point mutations in mtDNA. We characterized the cell lines in terms of heteroplasmy, respiration, and cell growth, and then performed a small scale “clinical trial in a dish” to assess the response of these cell lines to idebenone, a small molecule approved for Leber’s hereditary optic neuropathy (LHON).

## Main text

### Methods

#### Cell culture

LCLs were obtained from the Coriell Institute for Medical Research (Camden, NJ), see below. LCLs were cultured in RPMI (Corning 10-040) + 10% FBS (Seradigm 1500-500) + Antibiotic/Antimycotic (Gibco 15240062) at 37 °C and 5% CO_2_.

**Table:** 

Lymphoblastoid cell line	mtDNA mutation	Effect of mutation	Disease	Sex	Age at sampling (years)	Race
GM00333 (wildtype)	n/a	n/a	None	Female	23	Caucasian
GM11605 (ND1_3460G>A)	3460G>A; lies in mtND1	Alanine to threonine	LHON	Female	40	Caucasian
GM10742 (ND4_11778G>A)	11778G>A; lies in mtND4	Arginine to histidine	LHON	Male	30	Caucasian
GM10744 (ND4_11778G>A)	11778G>A; lies in mtND4	Arginine to histidine	LHON	Male	53	Black
GM13741 (ATP6_8993T>G)	8993T>G; lies in mtATP6	Leucine to arginine	Leigh’s	Male	2	Other
GM13740 (ATP6_8993T>G)	8993T>G; lies in mtATP6	Leucine to arginine	Leigh’s	Male	12	Caucasian

#### ARMS qPCR

Total DNA from passage 7 LCLs were isolated using the DNeasy Blood & Tissue Kit (Qiagen 69504) and stored at − 20 °C. qPCR was carried out on the QuantStudio 7 Flex Real-Time PCR System using SYBR green master mix (ThermoFisher 4367659), primer pairs (below), and total DNA as a template. Cycle threshold (Ct) values were obtained using the QuantStudio 7 Software. Identical results were seen in passage 15 LCLs, indicating that the heteroplasmy levels did not spontaneously change while in culture (data not shown).

**Table:** 

Target	Forward primer	Reverse primer
mtND1 wildtype	CTACTACAACCCTTCGCTGAAG	GAGCGATGGTGAGAGCTAAGG
mtND1_3460G>A	CTACTACAACCCTTCGCTGAAA	AGAAGAGCGATGGTGAGAGC
mtND4 wildtype	CTACGAACGCACTCACAGTAG	AGGTTAGCGAGGCTTGCTAG
mtND4_11778G>A	CTACGAACGCACTCACAGTAA	AGGTTAGCGAGGCTTGCTAG
mtATP6 wildtype	TACTCATTCAACCAATAGCCAT	AAGTGTAGAGGGAAGGTTAATGG
mtATP6_8993T>G	TACTCATTCAACCAATAGCCAG	TTAGGTGCATGAGTAGGTGGC

#### Cell growth on glucose or galactose media

LCLs were washed with PBS and then resuspended in either glucose or galactose media at 2 × 10^5^ cells/ml. Glucose media: glucose-replete RPMI (Corning 10-040) + 10% FBS + Antibiotic/Antimycotic. Galactose media: glucose-free RPMI (Corning 10-043) + 10% FBS + Antibiotic/Antimycotic + 25 mM galactose (Sigma G0750) + 1 mM pyruvate (Gibco 11360-070) + 1 mM l-glutamine (Gibco 25030-081) + 50 µg/ml uridine (Sigma U3003). At multiple time points up to 6 days after cell seeding, cell number and viability was counted with the Viacount Kit (Millipore 4000-0041). Ratios were calculated by dividing the fold change in cell number in galactose media by the fold change in cell number in glucose media.

#### Oxygen consumption assay

LCLs were plated at 1 × 10^5^ cells/well in XF Base Media (Agilent 103334-100) + 10 mM glucose (ThermoFisher A24940-01) + 1 mM pyruvate + 1 mM l-glutamine in XFp cell culture miniplates (Agilent 103025-100) and centrifuged at 300×*g* for 5 min. The Seahorse XF Cell Mito Stress Test Kit (Agilent 103015-100) was performed according to manufacturer’s instructions on the Seahorse XFp Analyzer. In experiments involving idebenone, 1% DMSO was used as a control.

#### Statistical methods

Quantitative data shown in figures and tables are derived from at least three independent experiments. T-test or Dunnett’s multiple comparisons test after one-way ANOVA was performed using Microsoft Excel or Graphpad Prism 7 to determine p-values; a p-value below 0.05 was considered significant.

### Results

Six different LCLs were obtained: one from an apparently-healthy individual, one from a patient with the 3460G>A point mutation mtND1, two from patients with the 11778G>A point mutation in mtND4, and two from patients with the 8993T>G point mutation in mtATP6. The allele refractory mutation system (ARMS)-based quantitative PCR (qPCR) method was used to calculate the heteroplasmy, or percentage of mutant mtDNA, of the LCLs [4, 5]. All of the mitochondrial disease associated LCLs contained > 98% mutant mtDNA, whereas the LCLs from an unaffected individual (GM00333 (wildtype)) has ≤ 0.13% mutant mtDNA (Table 1).

**Table 1:** Calculation of heteroplasmy of LCLs

Panel (A)		
GM00333 (wildtype)	Ct(wildtype) − Ct(mutant)	% mutant mtDNA
3460G>A (ND1)	− 9.92 ± 0.08	0.1 ± 0.01
11778G>A (ND4)	− 10.47 ± 0.15	0.07 ± 0.01
8993T>G (ATP6)	− 9.93 ± 0.48	0.13 ± 0.04

Panel (B)		
Strain	Ct(wildtype) − Ct(mutant)	% mutant mtDNA
GM11605 (ND1_3460G>A)	12.42 ± 0.45	99.98 ± 0.01
GM10742 (ND4_11778G>A)	8.75 ± 0.57	99.71 ± 0.06
GM10744 (ND4_11778G>A)	8.99 ± 0.05	99.8 ± 0.01
GM13741 (ATP6_8993T>G)	6.94 ± 0.61	98.81 ± 0.39
GM13740 (ATP6_8993T>G)	7.76 ± 0.55	99.37 ± 0.2
GM11605:GM00333 (1:1)	0.7 ± 0.27	61.64 ± 4.46
GM11605:GM00333 (1:5)	− 2.05 ± 0.2	19.61 ± 2.16

(A) ARMS-qPCR was used to quantify wildtype or mutant forms of mtDNA in the 3 loci shown using total DNA isolated from GM00333 (wildtype) LCLs. Heteroplasmy was calculated based on Ct values. GM00333 (wildtype) cells have < 0.13% mutant mtDNA in either of the loci analyzed

(B) Similarly, heteroplasmy was calculated for LCLs known to harbor mtDNA mutations at the loci indicated. As a control, DNA isolated from GM11605 or GM00333 were mixed in the ratios shown prior to qPCR, and yields expected heteroplasmy values

Average ± SEM for three independent experiments are shown

**Fig. 1:**
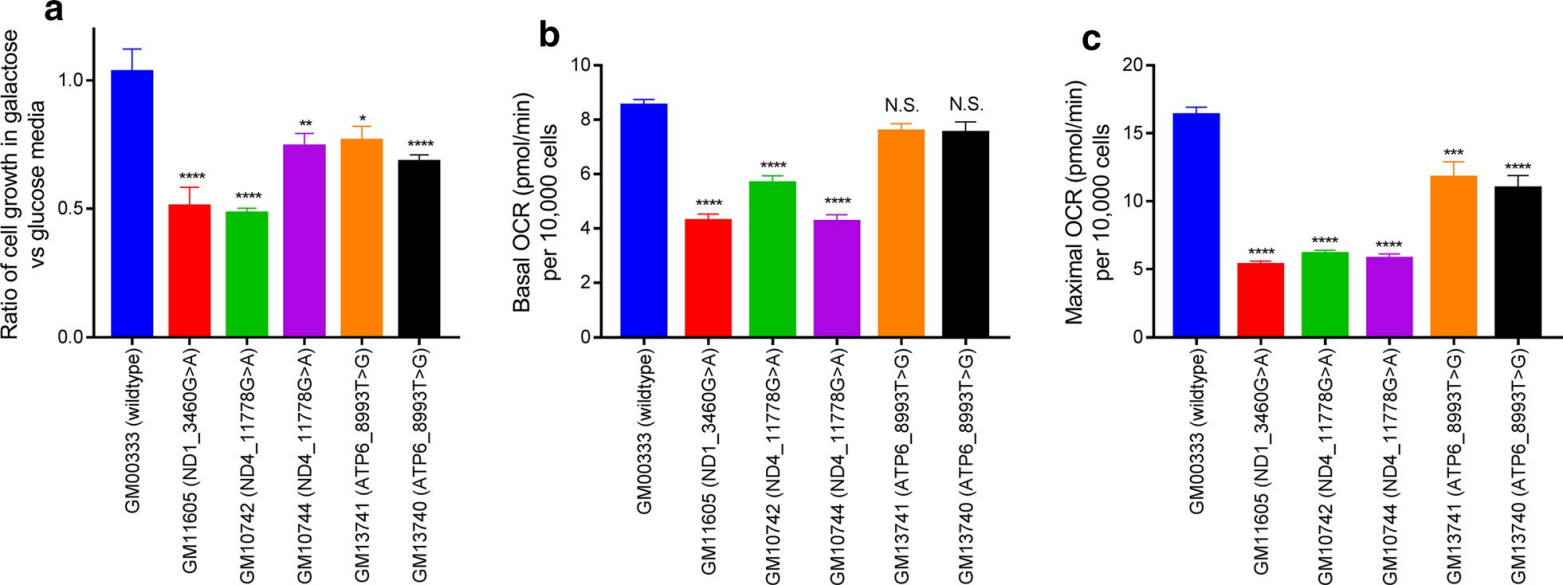
LCLs harboring mtDNA mutations display growth and respiration defects. **a** The ratios of cell growth in galactose media compared to glucose media are graphed. A ratio of 1.0 means that the cell line grows at the same rate in glucose or galactose media, whereas a ratio < 1.0 means that the cell line grows slower in galactose media compared to glucose media. GM00333 (wildtype) cells grow at a similar rate in either glucose or galactose media, but all LCLs with mtDNA mutations exhibit slower growth in galactose media. **b**, **c** Cells were seeded at equal confluency and oxygen consumption rates (OCR) were measured and plotted. Basal OCR is measured without the addition of mitochondrial inhibitors (**b**); maximal OCR is measured after the addition of oligomycin and FCCP (**c**). LCLs with mutations in complex I subunits display decreased basal respiration rates. All LCLs with mtDNA mutants display decreased maximal respiration rates. Average ± SEM from three independent experiments are shown. *p < 0.05, **p < 0.01, ***p < 0.001, ****p < 0.0001, by Dunnett’s multiple comparisons test after one-way ANOVA, vs. GM00333 (wildtype) cells

The 3460G>A, 11778G>A, and 8993T>G point mutations in mtDNA have previously been shown to decrease the function of their respective complexes [2, 6–8], leading to mitochondrial dysfunction. The ability of cells to grow in galactose media (i.e., glucose-free, galactose-replete media) has been widely used as a method to assess mitochondrial dysfunction [9]. Since the metabolism of galactose yields zero net ATP from glycolysis, cells grown in galactose media are forced to rely on oxidative phosphorylation for ATP production. Therefore, cells with mitochondrial dysfunction do not grow as well in galactose media as they do in glucose-replete media. We compared the ability of the LCLs to grow in either glucose or galactose media. While GM00333 (wildtype) cells grow at a similar rate in either glucose or galactose media, the LCLs carrying mtDNA mutations grow slower in galactose media compared to glucose media (Fig. 1a). Our results show that the presence of the mtDNA mutations cause mitochondrial dysfunction, and is consistent with previously published data [10, 11].

The measurement of respiratory rates in cells is another widely used method to assess mitochondrial dysfunction [5, 12]. Mitochondria are responsible for the majority of oxygen consumption occurring in the cell. Previously, it has been shown that lymphocytes respond to the addition of mitochondrial modulators in whole cell respiration studies [3]. We measured the oxygen consumption rates (OCR) of intact LCLs at basal levels and in response to the addition of oligomycin and carbonyl cyanide 4-(trifluoromethoxy) phenylhydrazone (FCCP). LCLs containing the ND1_3460G>A or ND4_11778G>A point mutation have a lower basal and maximal OCR than GM00333 (wildtype) cells (Fig. 1b, c). On the other hand, LCLs with the 8993T>G mutation in mtATP6 only have a lower maximal respiration. Both of these findings are consistent with previously published results regarding these point mutations [2, 13].

Idebenone is a cell permeable analog of coenzyme Q that has been approved for the treatment of LHON in Europe. Ninety to 95% of LHON cases are associated with point mutations in either ND1_3460G>A, ND4_11778G>A, or ND6_14484T>C [14–16]. In the cell, idebenone is reduced to idebenol by complex I or II of the electron transport chain (ETC), mitochondrial glycerol-3-phosphate dehydrogenase, or cytosolic NAD(P)H quinone oxidoreductase 1 [17]. Idebenol is then oxidized back to idebenone by complex III of the ETC. Thus, idebenone can bypass complex I and provide electrons directly to complex III of the ETC. Idebenone is also known to be a powerful antioxidant [18]. Idebenone has been shown to boost basal respiration in intact cells independently of complex I in at least two different mitochondrial disease models [17, 19].

**Fig. 2:**
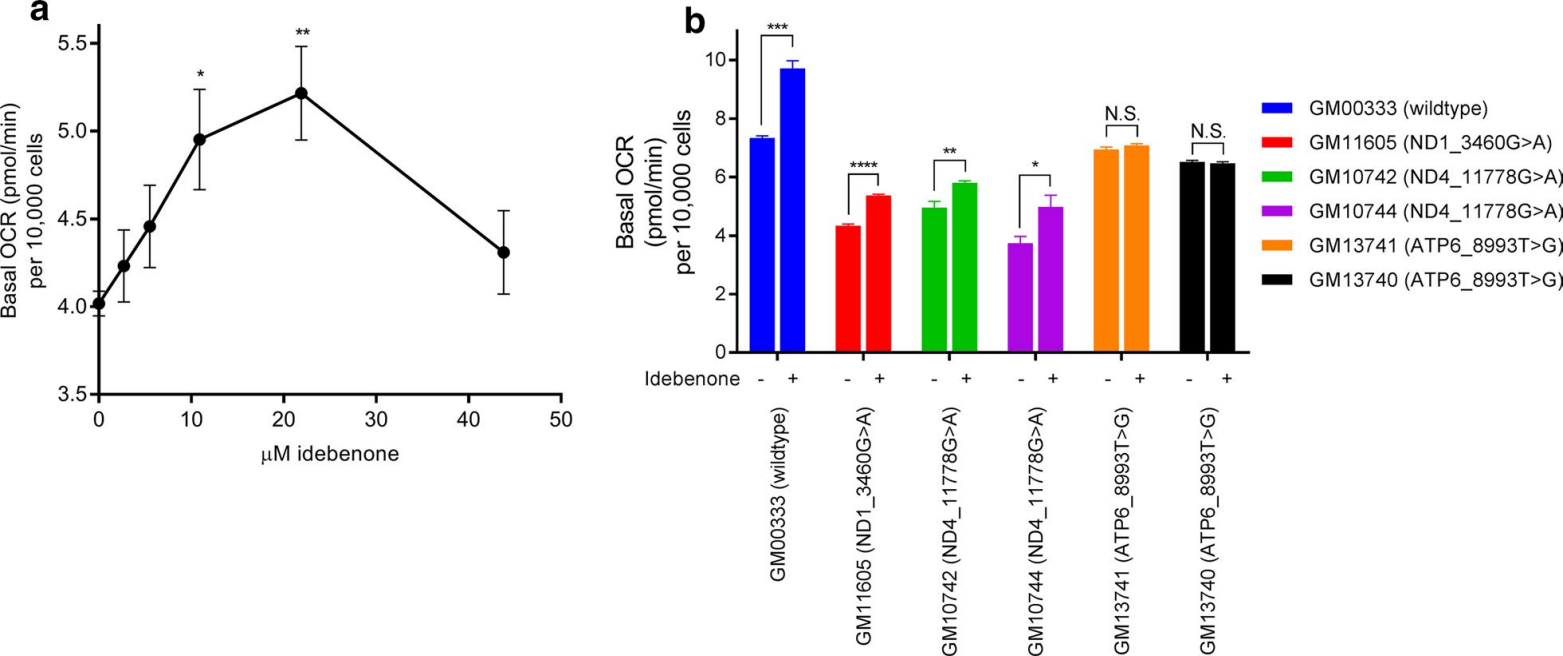
Idebenone increases the basal respiration of some, but not all, LCLs. **a** Idebenone increased the basal OCR of GM11605 (ND1_3460G>A) cells in a dose dependent manner. **b** Idebenone increased the basal OCR of wildtype LCLs and LCLs harboring complex I mutations. No effect was seen in LCLs harboring the 8993T>G mutation. Average ± SEM from three independent experiments are shown. *p < 0.05, **p < 0.01, ***p < 0.001, ****p < 0.0001, unpaired t-test, vehicle vs idebenone treated

Similarly, we found that idebenone increased the respiration of GM11605 (ND1_3460G>A) cells in a dose dependent manner (Fig. 2a), with 21.9 µM idebenone producing the maximal increase in OCR. We then tested the effect of 21.9 µM idebenone on basal respiration in all six LCLs. In either the wildtype LCLs or LCLs containing ND1_3460G>A or ND4_11778G>A mutations, idebenone increased the basal respiration of the cells (Fig. 2b). The idebenone-induced increase in respiration is sensitive to oligomycin and abolished with the addition of rotenone/antimycin (data not shown), as seen in other studies [17, 19]. Interestingly, however, we found that idebenone could not increase the basal respiration of cells harboring the ATP6_8993T>G mutation. To our knowledge, this is the first report of a lack of efficacy of idebenone in cells harboring the ATP6_8993T>G mutation. Our data suggests that the 8993T>G point mutation may confer resistance to idebenone and has implications for the clinical efficacy of idebenone in specific populations.

### Discussion

LCLs are an excellent model of mitochondrial disease that can be easily characterized in high-throughput, functional assays. A collection of LCLs from different patients are a useful tool that can be used to measure the efficacy of small molecules in vitro. Such “clinical trial in a dish” studies can expedite the discovery of broadly-applicable small molecule therapeutics for mitochondrial disease, and/or identify genetic mutations that may confer resistance to such therapies. Besides the “clinical trial in a dish” studies described in this paper, LCLs can also be used in personalized medicine applications. For example, LCLs can be generated from a specific patient, and libraries of small molecules can be screened for therapeutic effect in vitro. LCL based studies can also be used as a companion diagnostic for existing small molecule therapeutics. We conclude that LCLs are a flexible in vitro model of mitochondrial disease that can be used for a variety small molecule studies. We hope that our results will facilitate the discovery of new, broadly-applicable small molecule therapeutics for mitochondrial disease.

## Limitations

- The mechanism by which the 8993T>G mutation confers resistance to idebenone is unknown at this time.
- The number of cell lines used for analysis and comparison are small. Further studies using more LCLs and more assays can strengthen the claim that the 8993T>G mutation in mtATP6 provides resistance to idebenone.
- Ideally, a clinical trial in a dish study would compare hundreds of different LCLs in a 96 well plate format. The respiration and galactose growth assays can both be done in 96 well plates. This paper describes a pilot study done on only six different LCLs.

## Abbreviations

mtDNA: mitochondrial DNA
LCLs: lymphoblastoid cell lines
LHON: Leber’s hereditary optic neuropathy
ARMS-qPCR: allele refractory mutation system-based quantitative polymerase chain reaction
OCR: oxygen consumption rate
FCCP: carbonyl cyanide 4-(trifluoromethoxy)phenylhydrazone
ETC: electron transport chain

## References

[1] Gorman GS, Schaefer AM, Ng Y, Gomez N, Blakely EL, Alston CL (2015). Prevalence of nuclear and mitochondrial DNA mutations related to adult mitochondrial disease: adult mitochondrial disease. Ann Neurol.

[2] Brown MD, Trounce IA, Jun AS, Allen JC, Wallace DC (2000). Functional analysis of lymphoblast and cybrid mitochondria containing the 3460, 11778, or 14484 Leber’s hereditary optic neuropathy mitochondrial DNA mutation. J Biol Chem.

[3] Chacko BK, Kramer PA, Ravi S, Johnson MS, Hardy RW, Ballinger SW (2013). Methods for defining distinct bioenergetic profiles in platelets, lymphocytes, monocytes, and neutrophils, and the oxidative burst from human blood. Lab Invest.

[4] Venegas V, Halberg MC (2012). Quantification of mtDNA mutation heteroplasmy (ARMS qPCR). Methods Mol Biol Clifton NJ..

[5] Boominathan A, Vanhoozer S, Basisty N, Powers K, Crampton AL, Wang X (2016). Stable nuclear expression of ATP8 and ATP6 genes rescues a mtDNA Complex V null mutant. Nucleic Acids Res..

[6] Tatuch Y, Robinson BH (1993). The mitochondrial DNA mutation at 8993 associated with NARP slows the rate of ATP synthesis in isolated lymphoblast mitochondria. Biochem Biophys Res Commun.

[7] Tatuch Y, Pagon RA, Vlcek B, Roberts R, Korson M, Robinson BH (1994). The 8993 mtDNA mutation: heteroplasmy and clinical presentation in three families. Eur J Hum Genet EJHG..

[8] Trounce I, Neill S, Wallace DC (1994). Cytoplasmic transfer of the mtDNA nt 8993 T-->G (ATP6) point mutation associated with Leigh syndrome into mtDNA-less cells demonstrates cosegregation with a decrease in state III respiration and ADP/O ratio. Proc Natl Acad Sci USA..

[9] Gohil VM, Sheth SA, Nilsson R, Wojtovich AP, Lee JH, Perocchi F (2010). Nutrient-sensitized screening for drugs that shift energy metabolism from mitochondrial respiration to glycolysis. Nat Biotechnol.

[10] Bonnet C, Kaltimbacher V, Ellouze S, Augustin S, Bénit P, Forster V (2007). Allotopic mRNA localization to the mitochondrial surface rescues respiratory chain defects in fibroblasts harboring mitochondrial DNA mutations affecting complex I or v subunits. Rejuvenation Res..

[11] Bonnet C, Augustin S, Ellouze S, Bénit P, Bouaita A, Rustin P (2008). The optimized allotopic expression of ND1 or ND4 genes restores respiratory chain complex I activity in fibroblasts harboring mutations in these genes. Biochim Biophys Acta.

[12] Smolina N, Bruton J, Kostareva A, Sejersen T (2017). Assaying mitochondrial respiration as an indicator of cellular metabolism and fitness. Methods Mol Biol Clifton NJ..

[13] Nijtmans LGJ, Henderson NS, Attardi G, Holt IJ (2001). Impaired ATP synthase assembly associated with a mutation in the human ATP synthase subunit 6 gene. J Biol Chem.

[14] Wallace DC, Singh G, Lott MT, Hodge JA, Schurr TG, Lezza AM (1988). Mitochondrial DNA mutation associated with Leber’s hereditary optic neuropathy. Science.

[15] Huoponen K, Vilkki J, Aula P, Nikoskelainen EK, Savontaus ML (1991). A new mtDNA mutation associated with Leber hereditary optic neuroretinopathy. Am J Hum Genet.

[16] Kirches ELHON (2011). Mitochondrial Mutations and More. Curr Genomics.

[17] Vafai SB, Mevers E, Higgins KW, Fomina Y, Zhang J, Mandinova A (2016). Natural product screening reveals naphthoquinone complex I bypass factors. PLoS ONE..

[18] Mordente A, Martorana GE, Minotti G, Giardina B (1998). Antioxidant properties of 2,3-dimethoxy-5-methyl- 6-(10-hydroxydecyl)-1,4-benzoquinone (idebenone). Chem Res Toxicol.

[19] Giorgio V, Petronilli V, Ghelli A, Carelli V, Rugolo M, Lenaz G (2012). The effects of idebenone on mitochondrial bioenergetics. Biochim Biophys Acta BBA Bioenerg.

